# Effect of BMI and Perceived Importance of Health on the Health Behavior of College Students: Cross-Sectional Study

**DOI:** 10.2196/17640

**Published:** 2020-06-11

**Authors:** Wan-Chen Hsu, Chia-Hsun Chiang

**Affiliations:** 1 National Kaohsiung University of Sciences and Technology Kaohsiung Taiwan; 2 National Sun Yat-Sen University Kaohsiung Taiwan

**Keywords:** body mass index, college students, health behavior, perceived importance of health

## Abstract

**Background:**

Both body mass index (BMI) and the perceived importance of health have received a lot of attention, but few studies have fully investigated the interaction of their effects on health behaviors.

**Objective:**

This study investigates the effects of BMI and the perceived importance of health on health behaviors (patterns of eating, sleeping, and exercising) among college students in Taiwan.

**Methods:**

A survey was conducted with 334 students to assess their perception of the importance of health (using indicators) and their health behavior (using the Health Behaviors Scale). Respondent BMI was calculated from self-reported body weight and height. Descriptive statistical analysis, independent t test analysis, two-way analysis of variance (ANOVA), and one-way ANOVA were conducted.

**Results:**

The results showed a significant difference between genders in health behaviors among college students (eating: *t*_332_=2.17, *P*=.03; exercise: *t*_332_=5.57, *P*<.001; sleep: *t*_332_=2.58, *P*=.01). Moreover, there was an interaction between BMI and perception (of the importance of health) for exercise behaviors (*F*_2,328_=3.50, *P*=.03), but not for eating behaviors (*F*_2,328_=0.12, *P*=.89) or sleep behaviors (*F*_2,328_=1.64, *P*=.20).

**Conclusions:**

This study establishes, for the first time, the interaction of BMI and the perceived importance of health on health behaviors. The perception of health was found to have a significant effect on exercise behaviors. Thus, the perception of health plays a significant role in the exercise behaviors of college students in Taiwan. This finding provides researchers, policy makers, and practitioners with evidence, and consequently, opportunities for focusing on preventive actions. The findings suggest that increasing the importance of health in the perception of college students, should be the focus of efforts to help students exercise more regularly.

## Introduction

Both identity and health habits are formed during a young person’s transition from adolescence to adulthood [1]. The period during which one attends college is the ideal time to establish positive healthy behavior and eliminate unhealthy ones [2]. Although these years provide personal freedom and opportunities for new experiences, there is also the potential to adopt unhealthy and risky behavior [3]. Common health issues that affect young people at the start of college are poor sleep quality and lack of sleep [4]. In particular, students in the first year of college face several stressors and experience changes in their dietary and exercise patterns [5]. In its 2018 annual report, Taiwan’s Health Promotion Administration highlighted the increase in the number of overweight and obese students over 18 years of age [6]. The findings of their Changes in National Nutrition and Health Survey suggest the need for a deeper understanding of the health behaviors of Taiwanese college students [6].

Healthy behavior, such as making good dietary choices, exercising regularly, and maintaining regular sleep patterns, consists of positive actions that maintain or enhance health [7]. In general, healthy eating, sleeping, and exercise habits are each related to individual factors [8]. Scholars have argued that the perception that health is important prompts an individual to adopt a health-promoting lifestyle [9]. Previous studies have shown that individuals who place the utmost importance on health tend to adopt healthy behavior with respect to diet and nutrition, exercise [7-9], and sleep [7,8].

The link between body mass index (BMI) and health behaviors has also been the focus of research. Diet-related behaviors [10], dietary fat intake, amount of physical activity, amount of sedentary leisure time [11], and unhealthy behaviors [12] were all found to be associated with BMI. Students with a low BMI were less likely to snack and more likely to eat breakfast regularly [13]. In contrast, a high BMI was associated with unhealthy dietary patterns [14], eating irregularly [15], or eating a diet low in fiber-density [16]. Obesity and being overweight have been associated with being less likely to exercise regularly [17]. A U-shaped curvilinear association was found between sleep duration and BMI [18]—being overweight [19] or being underweight [20] was associated with poor sleep quality.

The roles of BMI and the perceived importance of health have received a lot of attention, but few studies have fully investigated the interaction of their effects on health behaviors. Because an in-depth study of these aspects would be useful in guiding the development of an effective educational program that promotes healthy behavior among college students, the aim of this study was to investigate the interaction of their effects. We hypothesize that there is an interaction between the effects of the perceived importance of health and BMI on the health behaviors of college students. Additionally, we hypothesize there is a difference between male and female health behaviors. Gender has been found to be a strong predictor of health behaviors [21] and studies have shown that female college students are more likely than male college students to have poor eating habits [22,23], poor exercise habits [23], and poor sleep habits [8].

## Methods

### Respondents

A questionnaire was administered to 360 college students from 12 colleges of which 334 valid questionnaires were returned (334/360, 92.8%). Of the total respondents, 50.0% were male (167/334) and 50.0% were female (167/334); 47.9% (160/334) attended college in the northern region, 23.1% (77/334) attended college in the central region, and 29.0% (97/334) attended college in the southern region of Taiwan; and the mean age was 20.70 (SD 1.35) years.

### Questionnaire

Data collected through the questionnaire included age, gender, height, weight, an item indicating perceived importance of health, and the 12-item Health Behaviors Scale. Using the reference standard of Taiwan’s Ministry of Health and Welfare [24], respondents were classified into three groups according to their BMI: values less than 18.5, values under 24 and greater than or equal to 18.5, and values greater than or equal to 24.

To assess respondents’ perceived importance of health, we employed an item designed by Beşer et al [9]. The degree of importance assigned to health was measured by a 5-point Likert scale ranging from “none” (coded as 1) to “very much” (coded as 5) in response to the question: “To what degree do you give importance to your health?” [9]. To avoid group divisions that were not meaningful, respondents were classified into two groups according to their scores: scores less than or equal to 3 (low importance) and scores greater than or equal to 4 (high importance).

The respondents’ health behaviors were evaluated using Chiang et al’s [8] Health Behaviors Scale which consists of 12 items that address three aspects of health behavior: exercise (four items), eating (four items), and sleep behaviors (four items). Using item analysis, exploratory factor analysis, and confirmatory factor analysis, Chiang et al [8] showed that the Health Behaviors Scale is a valid and reliable measure of health behaviors for Taiwanese college students (Cronbach α=.83). Respondents answered the 12-item Health Behaviors Scale on a 5-point Likert scale with scores ranging from 1 (never) to 5 (always). Higher scores indicated that respondents have regular physical activity and exercise, healthy eating habits, healthy sleep habits, and good quality sleep. In terms of internal consistency, the exercise (Cronbach α=.79), eating (Cronbach α=.77), and sleep (Cronbach α=.80) showed good reliability.

### Data Analysis

Statistical analysis was performed using SPSS software (version 20.0; IBM Corp). First, for a better understanding of the respondents’ characteristics, descriptive statistical analysis was performed. Second, an independent *t* test (two-tailed) was used to identify if a gender difference in health behaviors existed. A *P* value less than .05 indicated that there was a statistically significant difference between genders. Finally, a two-way analysis of variance (ANOVA) was used to determine the effects of BMI and perceived importance of health on exercise, eating, and sleep behaviors among respondents. If the interaction of BMI and the perceived importance of health was significant (*P*<.05), then a one-way ANOVA was performed to assess the simple effects of the perceived importance of health and BMI.

## Results

### Descriptive Statistics

Table 1 presents a descriptive summary of each health behavior for the corresponding perceived importance of health and BMI groups. The mean scores were 13.46 (SD 3.06) for eating behaviors, 13.75 (SD 3.61) for exercise behaviors, and 10.66 (SD 3.56) for sleep behaviors.

**Table 1:** Descriptive summary for each health behavior by perceived importance of health and BMI.

Respondent groupings, n (%)			Health behavior scores		
			Eating, mean (SD)	Exercise, mean (SD)	Sleep, mean (SD)
**Low perception of health importance**		156 (100)	12.31 (2.98)	12.81 (3.41)	9.51 (3.21)
	BMI<18.5	21 (13.5)	11.38 (2.78)	11.86 (3.77)	8.05 (2.29)
	18.5≤BMI<24	88 (56.4)	12.32 (3.17)	13.25 (3.49)	9.57 (3.35)
	BMI≥24	47 (30.1)	12.72 (2.63)	12.43 (3.01)	10.06 (3.15)
**High perception of health importance**		178 (100)	14.47 (2.78)	14.57 (3.60)	11.67 (3.55)
	BMI<18.5	20 (11.2)	13.75 (2.10)	11.90 (2.40)	11.70 (2.96)
	18.5≤BMI<24	121 (68.0)	14.48 (2.66)	14.60 (3.62)	11.76 (3.69)
	BMI≥24	37 (20.8)	14.84 (3.40)	15.89 (3.32)	11.38 (3.43)
**All**		334 (100)	13.46 (3.06)	13.75 (3.61)	10.66 (3.56)
	BMI<18.5	41 (12.3)	12.54 (2.72)	11.88 (3.14)	9.83 (3.19)
	18.5≤BMI<24	209 (62.6)	13.57 (3.07)	14.03 (3.62)	10.84 (3.70)
	BMI≥24	84 (25.1)	13.65 (3.15)	13.95 (3.58)	10.64 (3.32)

### Gender Differences in Health Behaviors

Table 2 reveals gender differences in college students’ eating, exercise, and sleep behaviors indicating that male students have healthier eating behaviors (*P*=.03), better exercise habits (*P*<.001), and better sleep quality (*P*=.01) than female students.

**Table 2:** Health behavior scores showing gender differences.

Health behaviors	Gender, mean (SD)		*t* test (*df*)	*P* value
	Male (n=167)	Female (n=167)		
Eating	13.83 (3.10)	13.10 (2.99)	2.17 (332)	.03
Exercise	14.80 (3.69)	12.69 (3.21)	5.57 (332)	<.001
Sleep	11.16 (3.51)	10.17 (3.54)	2.58 (332)	.01

### Effects of BMI and Perceived Importance of Health on Eating, Exercise, and Sleep Behaviors

As shown in Table 3, for eating behaviors, there were statistically significant main effects for BMI (*F*_2,328_=3.66, *P*=.03) and for the perceived importance of health (*F*_1,328_=29.44, *P*<.001); however, the interaction effect between BMI and the perceived importance of health was not statistically significant (*F*_2,328_=.12, *P*=.89).

For exercise behaviors, there were statistically significant main effects for BMI (*F*_2,328_=6.91, *P*=.001) and for the perceived importance of health (*F*_1,328_=14.66, *P*<.001) as well as a statistically significant interaction between BMI and the perceived importance of health (*F*_2,328_=3.50, *P*=.03).

For sleep behaviors, there was a statistically significant main effect for the perceived importance of health (*F*_1,328_=26.99, *P*<.001); however, there was no statistically significant main effect for either BMI (*F*_2,328_=1.03, *P*=.36) or the interaction between BMI and the perceived importance of health (*F*_2,328_=1.64, *P*=.20).

As indicated by the simple effects analysis of the perceived importance of health and BMI on exercise behaviors shown in Table 4, respondents who considered health to be important and whose BMI was greater than or equal to 18.5 had significantly higher exercise behavior scores than respondents who did not consider health to be as important and whose BMI was greater than or equal to 18.5. In addition, respondents whose BMI was greater than or equal to 18.5 and who considered health to be important had significantly higher exercise behavior scores than respondents whose BMI was less than 18.5 and who considered health to be important.

**Table 3:** Statistical results of the two-way analysis of variance for the effects of perceived importance of health and BMI on health behaviors.

Health behaviors	BMI		Importance of health			BMI × Importance of health	
	*F* test (*df*)	*P* value	*F* test (*df*)	*P* value	*F* test (*df*)		*P* value
Eating	3.66 (2,328)	.03	29.44 (1,328)	<.001	0.12 (2,328)		.89
Exercise	6.91 (2,328)	.001	14.66 (1,328)	<.001	3.50 (2,328)		.03
Sleep	1.03 (2,328)	.36	26.99 (1,328)	<.001	1.64 (2,328)		.20

**Table 4:** Simple effects of the perceived importance of health and BMI on exercise behaviors.

Exercise behaviors scores, mean (SD)			Perception groups			Perception effect			
		Low		High		*F* test (*df*)		*P* value	
**BMI groups**									
	BMI<18.5	11.86 (3.77)		11.90 (2.40)^a,b^		0.002 (1,39)		.97	
	18.5≤BMI<24	13.25 (3.49)		14.60 (3.62)^a^		7.34 (1,207)		.007	
	BMI≥24	12.43 (3.01)		15.89 (3.32)^b^		25.03 (1,82)		<.001	
**BMI effect**									
	*F* test (*df*)	1.87 (2,153)		8.72 (2,175)		N/A^c^		N/A^c^	
	*P* value	.16		<.001		N/A^c^		N/A^c^	

^a^Indicates belonging to the a pair, and statistically significant difference between the pairings (*P*=.006)

^b^Indicates belonging to the b pair, and statistically significant difference between the pairings (*P*<.001).

^c^N/A: Not applicable.

## Discussion

### Principal Findings

This study aimed to identify the interaction the effects of BMI and perceived importance of health on eating, exercise, and sleep behaviors. Moreover, there were significant gender differences in health behaviors among Taiwanese college students. Finally, there was an interaction between the effects of BMI and perceived importance of health for exercise behaviors, but not for eating or sleep behaviors. The effect of the perceived importance of health on exercise behavior was only true for those with BMI greater than 18.5; for underweight individuals, the perceived importance of health had no effect on exercise behaviors. Thus, the research hypothesis was partly supported.

Previous studies have found that being overweight is associated with physical inactivity and sedentary daily habits [17]. Thus, overweight and obese students may require encouragement to undertake physical activity. This study found an interaction between the effects of BMI and the perceived importance of health on exercise behaviors. College students with normal and above normal BMI who placed importance on health were more likely to exercise regularly than those with below normal BMI. Consistent with previous studies [7-9], we found the perceived importance of health plays a significant role in adopting healthy exercise behavior. Therefore, increasing the perception of health among overweight students may encourage them to participate more in physical activities.

This study found that the mean score of sleep behaviors was 10.66 (range 4-20). Previous studies have shown that college students have poor sleep quality and irregular sleeping habits [8,25,26]. College students with higher stress levels experience poor sleep quality [25,26]. Many health education programs are focused on improving knowledge and skills related to regular exercise and a healthy diet. However, sleep quality and problems related to sleep are overlooked [27]. School and government authorities need to be more proactive in designing appropriate sleep and stress management strategies to help college students improve their sleep quality and maintain positive sleep habits.

This study showed that male students have healthier eating behaviors, better exercise habits, and better sleep quality than female students supporting the findings of previous studies [8,22,23]. These results suggest that gender-specific eating, exercise, and sleep intervention programs for college students are necessary.

Finally, this study found that the interaction between the effects of BMI and perceived importance of health did not affect health behaviors related to eating and sleeping; however, the study also revealed that the eating behaviors of college students with different BMI show significant differences but sleep behaviors do not. College students with different levels of perceived importance of health also have significant differences in their eating and sleeping behaviors. These findings reveal that college students’ eating behaviors are influenced by their BMI and both eating and sleeping behaviors are influenced by perceived importance of health.

### Limitations

The study sample consisted of respondents in a higher education setting and was restricted by age to college students and by place to Taiwan. Consequently, the findings should not be overgeneralized and must be interpreted with consideration of the sample’s homogeneity. Finally, the perceived importance of health was measured using only one item. Future studies should include respondents of different ages as well as develop further instruments to measure this construct (the perceived importance of health).

### Conclusions

This study found that the interaction between the effects of BMI and the perceived importance of health does not affect the health behaviors of eating and sleeping. Previous studies have shown that college students’ choice to major in medicine and their self-rated health were related to their health behaviors [7,8]. Future studies should consider the effects of the interactions of these individual factors as well as BMI on eating and sleeping behaviors, to investigate how best to help students adopt regular eating habits and improve their sleep quality.

This study established, for the first time, the interaction between the effects of BMI and the perceived importance of health on health behaviors. This finding provides researchers, policy makers, and practitioners in the field with evidence and opportunities for focusing on preventive action. The findings of the study suggest that increasing the perceived importance of health should guide efforts to help students adopt better exercise habits. There were also significant gender differences in health behaviors among college students suggesting that health education practitioners should design gender-specific health behavior intervention programs for college students.
